# Light adaptive image enhancement for improving visual analysis in intercropping cultivation

**DOI:** 10.3389/fpls.2025.1639016

**Published:** 2025-08-20

**Authors:** Wei Zhong, Wanting Yang, Yunfei Wang, Xiang Dong, Xiaowen Wang, Weidong Jia, Mingxiong Ou, Mingde Yan

**Affiliations:** ^1^ School of Agricultural Engineering, Jiangsu University, Zhenjiang, China; ^2^ School of Mechatronic Engineering, Taizhou University, Taizhou, China; ^3^ Chinese Academy of Agriculture Mechanization Sciences Group Co., Ltd., Beijing, China

**Keywords:** illumination compensation, intercropping, height difference, solar elevation angle, growth stage

## Abstract

Intercropping maize and soybean with distinct plant heights is a typical practice in diversified cropping systems, where shadows cast by taller maize plants onto soybean rows pose significant challenges for image based recognition. This study conducted experiments throughout the entire soybean–maize intercropping period to address illumination variation. Based on the height difference between crops, solar elevation angle, and light intensity at the top of the soybean canopy, an illumination compensation regression model was developed. The model was applied to correct soybean canopy images and compared against traditional enhancement methods, including histogram equalization, Multi-Scale Retinex (MSR), and gamma correction. Quantitative evaluation using peak signal-to-noise ratio (PSNR) showed the proposed method achieved 40.79 dB, indicating superior image quality. Furthermore, analysis of RGB and HLS channels revealed a consistent increase in brightness from left (darker) to right (brighter) across the images. Specifically, green channel values rose from 150-230 to 180-240, and overall RGB values exceeded 150, suggesting improved brightness and reduced local fluctuations. Brightness increased from 90-200 to 150-220, with the left region rising from 125 to 175. Finally, a comparison of channel-wise standard deviations among methods showed that the proposed algorithm exhibited lower variance in the green (G) and hue (H) channels, with favorable consistency across others. These results demonstrate the model’s effectiveness in achieving smoother brightness transitions, thereby enhancing image uniformity and mitigating the negative impact of uneven illumination on recognition tasks.

## 1 Introduction

Intercropping has been widely adopted due to its advantages in intensive land use and production efficiency (Kass, 1978; Andersen, 2025). It is a cultivation system in which two or more crops are simultaneously grown in rows or strips on the same land within a single growing season (MacLaren et al., 2023; Du et al., 2018). On average, intercropping can increase production efficiency by 20–30% and also contributes to weed suppression and a reduction in pests and diseases (Ren et al., 2019). In intercropping systems, taller crops intercept more sunlight, casting shadows on shorter crops and reducing their exposure to direct radiation. This shading effect substantially influences the morphological and physiological traits of the understory crops (Franklin, 2008; Raza et al., 2019). Shading alters the microclimate experienced by short-statured crops by reducing the infrared radiation they receive, thereby affecting light intensity (Wang et al., 2021a; Zhu et al., 2015). These changes trigger a series of responses in plant phenotypes and physiology (Fan et al., 2018; Hussain et al., 2019), influencing critical traits such as stem diameter, leaf thickness, leaf area index (LAI), photosynthetic capacity, aboveground biomass, yield, and plant height (Deng et al., 2021; Petrella et al., 2022).

Despite its agronomic advantages, the large scale adoption of intercropping has been constrained by mechanization challenges (Bybee-Finley and Ryan, 2018). Accurate recognition of crop traits is a prerequisite for mechanized operations, yet uneven shading in intercropped systems hampers reliable image based detection. Shading induced brightness variation reduces the quality of captured images and interferes with visual perception and object recognition. Image recognition technologies have increasingly been applied in agriculture for tasks such as plant protection (Routray et al., 2019), harvesting, and sowing (Wang et al., 2022; Feng et al., 2024). Applications extend across multiple crop species, including wheat, maize, and rice (Zhu et al., 2023; Wang et al., 2021b; Sun et al., 2018, 2021). However, during image acquisition, both the lighting conditions and the medium through which light propagates significantly affect image quality (Xu et al., 2023). To address low light image degradation, researchers have explored both hardware and software solutions. Specialized low light cameras have demonstrated strong performance, but their high cost and limited practicality hinder widespread use. On the software side, digital image processing techniques remain the primary method for image enhancement, though challenges such as color distortion and uneven brightness persist (Li et al., 2022). Traditional enhancement methods include histogram equalization, which redistributes pixel intensity to expand an image’s dynamic range and improve contrast (Coltuc et al., 2006; Lee et al., 2013b). Additionally, image enhancement models based on Retinex theory have been developed, though they often struggle to balance brightness recovery with dynamic range compression. To overcome this limitation, the multi-scale Retinex (MSR) method was introduced and further refined by Lee et al. (Lee et al., 2013a), improving its parameter adaptability. MSR decomposes an image into reflectance and illumination components and has served as the foundation for various subsequent methods (Guo et al., 2016; Li et al., 2018). Similarly, gamma correction adjusts brightness by applying a nonlinear transformation that approximates human visual perception, improving brightness uniformity, especially in darker regions (Huang et al., 2012).

In intercropping systems, the shading distance varies according to the height of taller crops and the solar elevation angle. However, current studies offer limited research on the relationship between shading distance and light intensity. Statistical models constructed based on the shading capacity of intercropping systems using regression analysis are often heavily influenced by actual measurement data. To simulate the shading capacity at various positions within the canopy, it is essential to establish a general model that directly quantify light intensity in intercropping systems. Factors such as crop canopy height, the differences in crop height, and the solar elevation angle are crucial in determining the light environment, Continuous measurements throughout the entire growing season are required to capture data across different periods for improved accuracy. Strip intercropping of maize and soybean is widely practiced in China; hence, this study focuses on soybean-maize intercropping as a test subject. In shading research, soybean, being a shade-intolerant species, frequently receives more attention. A statistical analysis conducted by Li et al. (2023) on soybean–maize intercropping systems revealed that the taller maize plants significantly obstruct direct solar radiation, thereby influencing the light availability for shorter soybean plants. Specifically, the shading ratio for soybean in the southernmost row was reported to range between 52.44% and 57.44%, indicating substantial light interception. Although light compensation models have been applied in fields such as underwater imaging, their adaptation to open field agricultural settings remains limited. This study bridges that gap by incorporating crop specific geometry and solar parameters into the illumination correction process, marking a novel interdisciplinary application of physics based enhancement strategies. This study aims to develop a color constancy algorithm capable of counteracting the effects of various adverse light sources, thereby obtaining an intrinsic image that reflects fundamental physical properties of the scene’s surface.

This study aims to directly quantify the relationship between light intensity and shading in maize-soybean intercropping systems. The research objectives are as follows: (1) to develop a quantitative shading model that accounts for the geometric relationship between canopy structure (the height difference between adjacent maize and soybean canopies) and the position of the sun; (2) to process the shaded areas of images using traditional methods and evaluate the resulting image quality; and (3) to establish an image processing algorithm tailored to both tall and short crops, by applying an illumination Compensation method to smooth lighting effects in the images.

## 2 Materials and methods

### 2.1 Experimental design and image acquisition

**Figure 1:**
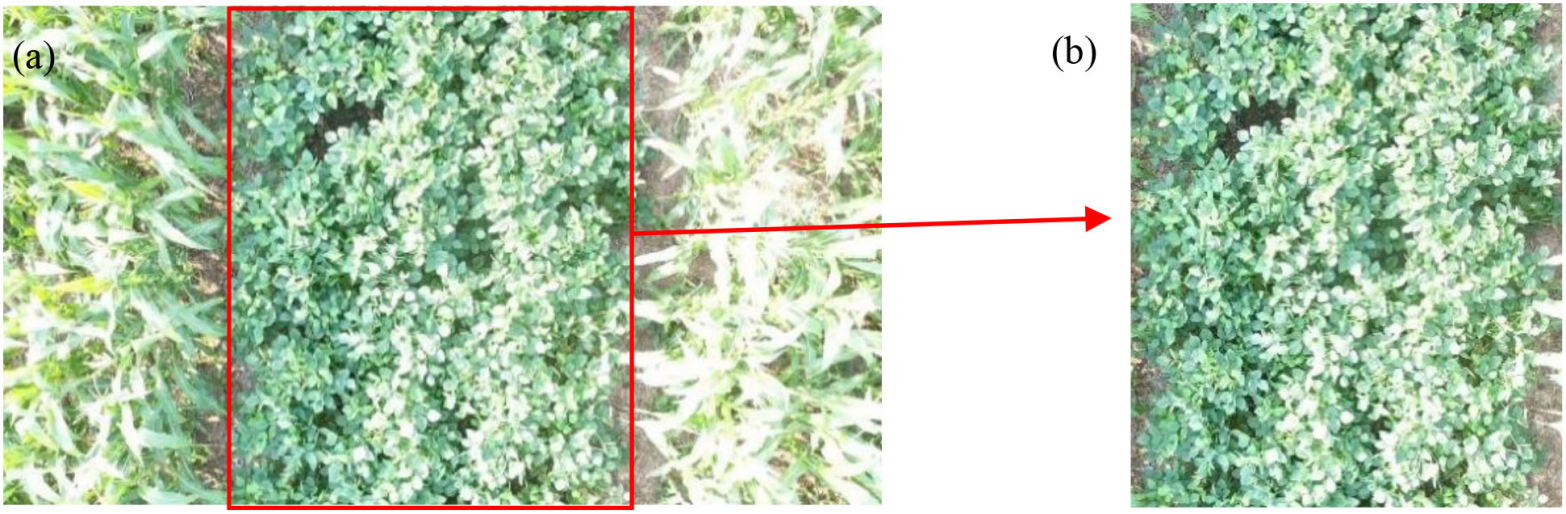
**(a)** Drone captured photos and **(b)** soybean strip photos.

A field experiment on soybean–maize intercropping was conducted at the Zhenjiang Agricultural Science and Technology Park in Jiangsu Province, China (32°12′ N, 119°18′ E). From 2020 to 2023, the site had an average annual temperature of 16.5°C, annual precipitation of 1105 mm, and annual sunshine duration of 1956 hours. On June 8, 2024, maize (Jiangyu 688) and soybean (Qihuang 34) were sown. The total experimental area was 6.67 ha, and the intercropping system consisted of four rows of maize (row spacing: 60 cm; plant spacing: 20 cm) alternated with six rows of soybean (row spacing: 30 cm; plant spacing: 10 cm), with a 60 cm wide buffer zone between the maize and soybean strips. The rows were oriented east–west to ensure consistent lighting conditions. Observations were carried out at five key growth stages of maize: the third leaf, seventh leaf, tasseling, milk, and maturity stages, which corresponded to the third leaf, flowering, pod setting, grain filling, and maturity stages of soybean, respectively. At each stage, plant height was measured, and light intensity at the top of the soybean canopy was recorded at 8:00 AM under clear sky conditions. In addition, images of the soybean strip were collected, as shown in **Figure 1**. Plant height was defined as the vertical distance from the stem base to the uppermost point of the canopy. For each growth stage, ten maize plants and ten soybean plants were randomly selected and measured. The average height for each species was calculated to represent the mean canopy height, and the difference in height between maize and soybean was used to assess shading effects. Light intensity measurements were taken at the top of each soybean row using a multi-light source illuminometer (DL333205, Deli Group, Ningbo, China). Three random positions per row were selected, and the average reading was used to represent the light intensity for each growth stage. Image acquisition was conducted using a DJI Mavic 2 drone (DJI, Shenzhen, China). A drone was employed to capture images from a height of 2 meters above the soybean canopy. The camera operated at a resolution of 2688 × 1512 pixels and was oriented vertically downward, with the flight direction aligned with the row orientation of the soybean plants. At each growth stage, images were collected from two adjacent plots: an experimental plot and a control plot. Specifically, 20 images per stage were taken from the experimental plot for model parameterization, while 10 images per stage were captured from the control plot to serve as a dataset for subsequent model validation. In this study, light intensity was measured across different soybean rows at various growth stages to obtain distribution profiles of illumination variation within the soybean strip. Based on these measurements, an illumination compensation algorithm was developed to address the uneven lighting conditions in intercropping systems. To validate the effectiveness of the proposed model, soybean canopy images were collected at multiple growth stages and processed using different enhancement algorithms. Comparative analyses of the RGB and HLS channel values before and after enhancement were conducted, allowing identification of the most effective image enhancement method.

### 2.2 Illumination compensation model construction

**Figure 2:**
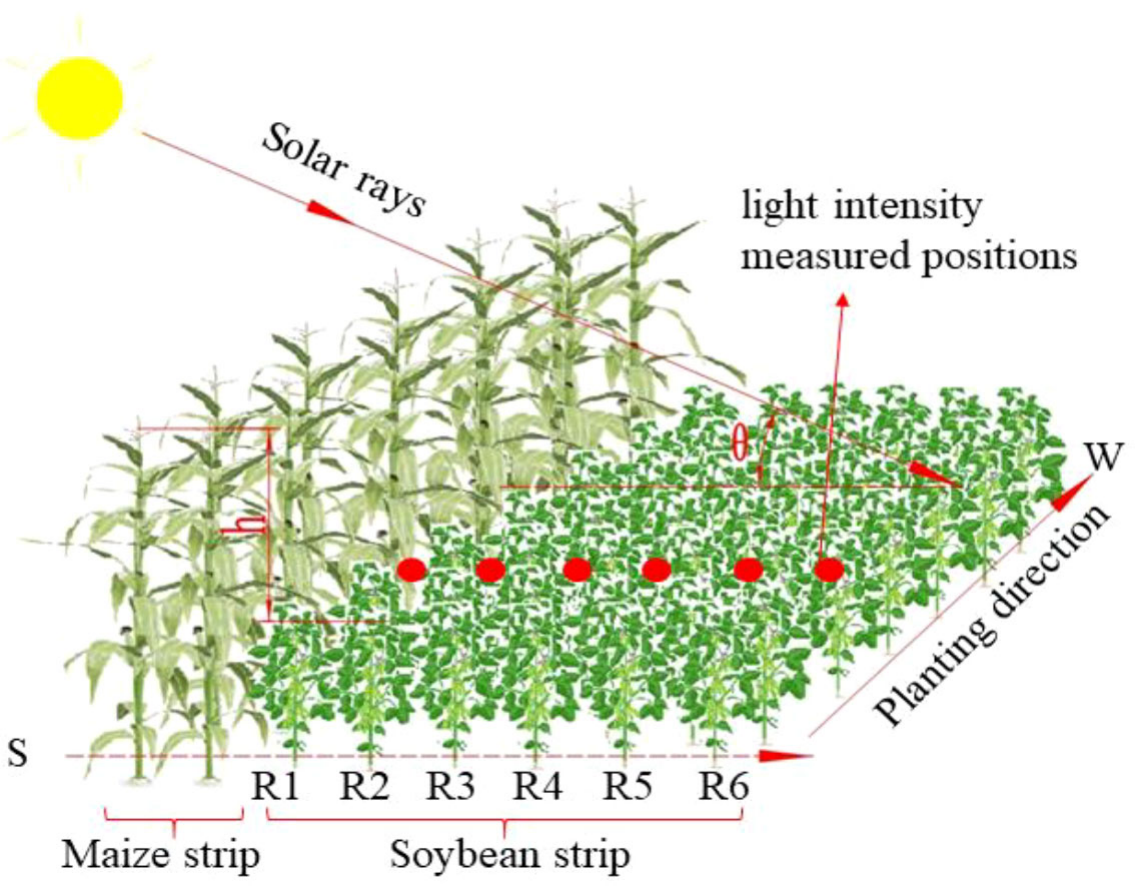
Schematic of the developed shading capacity mode.

In intercropping systems, uneven illumination within the soybean strip can negatively affect the accuracy of subsequent soybean information extraction. To address this issue, this study enhances soybean canopy images based on the observed variation in light intensity across different rows. This process is hereafter referred to as illumination compensation. In this study, the maize and soybean strips were simplified and modeled as rectangular cuboids. It was assumed that sunlight transmission through the maize canopy was negligible, and the light intensity received by the soybean strip was primarily influenced by direct solar radiation, atmospheric scattering, and reflected light from the adjacent maize strip to the south. The shading distance cast by the maize strip was determined primarily by plant height and the solar angle. However, there is limited research on the quantitative relationship between shading distance and irradiance. In this work, particular attention was given to the effects of plant height difference and solar elevation angle on shading behavior within the soybean strip. The height difference was defined as the vertical distance between the top of the maize canopy and the top of the soybean canopy, while the solar elevation angle referred to the angle between the sun’s rays and the horizontal plane. The experimental field was oriented east–west, and the soybean–maize intercropping system was observed from June to October, during which the soybean strip was primarily shaded by the southern maize strip throughout the day. To facilitate analysis, the soybean rows were labeled from south to north as R1 to R6, as shown in **Figure 2**.

Compensation curves reflecting the impact of plant height on light intensity across different growth stages were established through statistical analysis, yielding distinct light intensity curves for each stage. These curves were subsequently employed to enhance images by applying the corresponding light intensity adjustments. In the shaded areas, light intensity predominantly arises from direct sunlight, scattered light, and ambient reflected light. Moreover, the total light intensity in these regions is influenced by both the solar altitude angle and the height difference between the crops. Based on the Equations 1–6, the total illuminance in the shaded region was derived (Yan et al., 2015; Saraçoğlu and Gündüz, 2009).


(1) $$E=E_{0}\cos\text{θ}$$

Where E is the actual light intensity; *E_0_* is the light intensity of the sun in direct sunlight; *θ* is the solar elevation angle.


(2) $$L=\frac{h}{\tan\theta }$$

Where *L* is the length of the shade; *h* is the height difference of the crop.


(3) $$E_{shadow}=E_{0}\left(\cos\theta -f\left(h,d\right)\right)+E_{diffuse}+E_{reflected}$$

Where *E_shadow_* is the light intensity of the shaded area; f(h,d) is the shading effect due to the height difference h and the position d in the shadow; *E_diffuse_* is the scattered light, which usually accounts for 10-20% of the total radiation; and *E_reflected_* is the ambient reflected light.


(4) $$E_{diffuse}=E_{0}D_{f}$$

Where *D_f_* is the scattered light coefficient, 0.1-0.2 on a clear day.


(5) $$E_{reflected}=E_{0}\alpha$$

Where α is the reflectance, and the soybean line is 0.1.


(6) $$E_{shadow}=E_{0}\left(\cos\theta -f\left(h,d\right)\right)+E_{0}D_{f}+E_{0}\alpha$$

### 2.3 Illumination compensation algorithm

This section presents the illumination compensation algorithm (ICNet) developed in this study and compares its performance with three conventional enhancement methods: histogram equalization, multi-scale Retinex (MSR), and gamma correction. The comparison was conducted using both quantitative (PSNR) and qualitative (RGB/HLS) metrics. Histogram equalization enhances the overall contrast of an image by redistributing the grayscale values to achieve a more uniform intensity distribution. Specifically, it transforms the grayscale histogram using the cumulative distribution function, allowing the pixel values to be more evenly spread across the dynamic range. However, in images with substantial brightness differences, this method may lead to excessive noise amplification and structural distortion (Dai et al., 2019). Retinex is an image enhancement algorithm inspired by the human visual perception system, designed to separate the illumination and reflectance components of an image. The Multi-Scale Retinex (MSR) algorithm is an enhanced variant of the classical Retinex model, which estimates the illumination component at multiple spatial scales using Gaussian filtering and subsequently derives the reflectance by computing the logarithmic difference. This multi-scale approach enables the enhancement of image details across both low and high frequency regions, as formulated in Equation 7. Gamma correction is a nonlinear brightness adjustment technique that transforms image pixel values using a power law function to align with human visual sensitivity to brightness. When the gamma value γ<1, it enhances dark region details; when γ>1, it suppresses overexposed areas, as expressed in Equation 8.


(7) $$R_{i}\text{x},\text{y}=\log I_{i}\left(x,y\right)-\log[F_{i}\left(x,y\right)*I_{i}\left(x,y\right]$$

where $I_{i}$ denotes the original image, $F_{i}$ represents the Gaussian kernel at scale i , and * denotes the convolution operation.


(8) $$I_{out}=C\cdot I_{in}^{\gamma }$$

where γ is the gamma value, C is a constant, and $I_{in}$ and $I_{out}$ represent the input and output pixel values, respectively.

**Figure 3:**
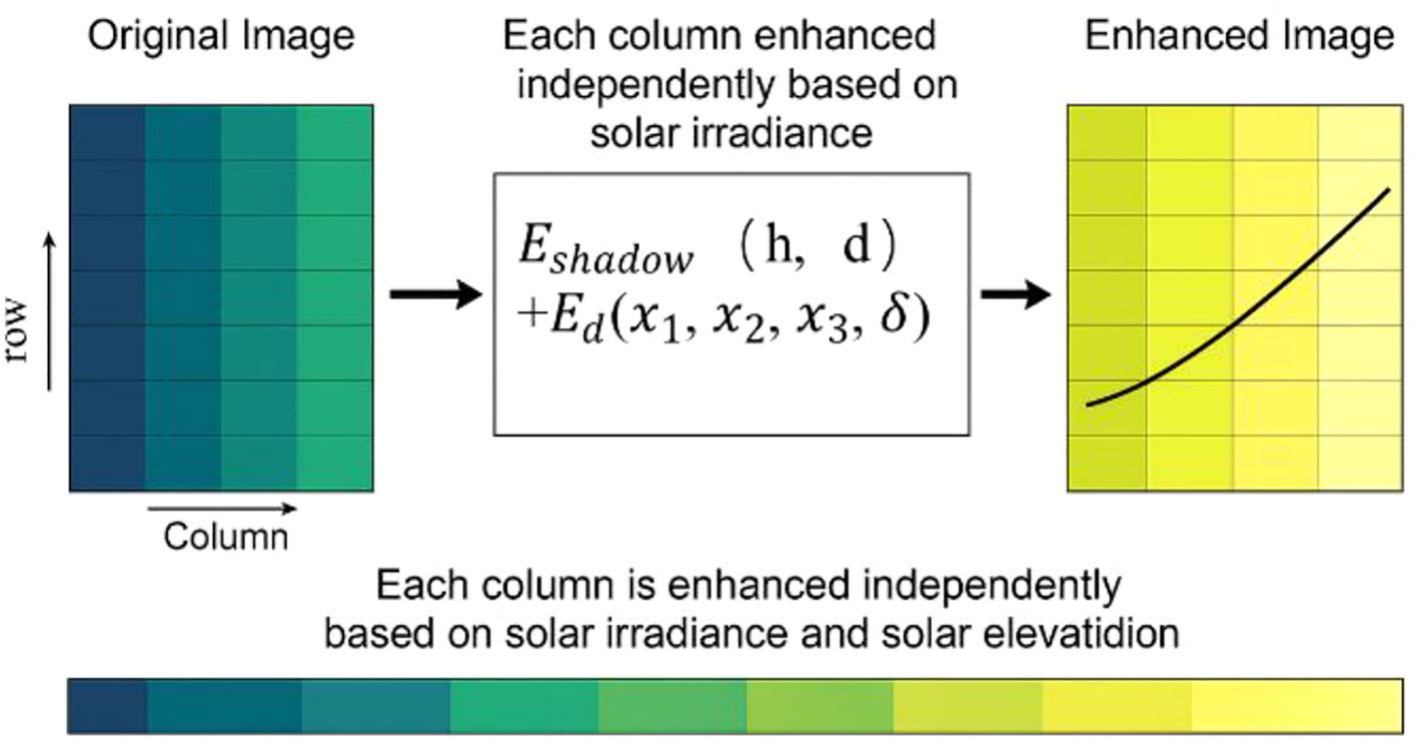
Column wise illumination enhancement based on fitted regression model.

Unlike conventional Retinex based methods that rely on generic assumptions of illumination distribution, ICNet incorporates physical field variables, including crop height difference and solar elevation angle, allowing for scene specific brightness adjustment. In this study, image data enhancement was performed using the aforementioned formulas by applying a compensation mapping to mitigate brightness variations and enhance overall image brightness. This algorithm is referred to as ICNet. To address the image quality issues arising from mutual shading between soybean and maize in intercropping systems, a novel algorithm for image brightness correction is proposed. The method adjusts brightness on a column by column basis to achieve uniform lighting across the image. Using a regression model, the target illumination intensity for each column of the image was calculated. Then, with a preset reference illumination intensity as a benchmark, the soybean rows (30, 60, 90, 120, 150, and 180 cm) were used as positional input coordinates. Illumination compensation was subsequently applied from left to right along the soybean belt to enhance overall image brightness. For each column, the adjustment ratio is calculated based on the target and reference light intensity, gradually aligning the brightness with the predetermined standard. To prevent excessive enhancement or reduction, the adjustment ratio is constrained within a range of 0.5 to 1.2. This ratio is then applied to the pixel values of each column to adjust the overall brightness. Finally, the processed image is normalized to the standard display range, thereby enhancing the accuracy of the enhancing image information, as shown in **Figure 3**.

### 2.4 Evaluation metrics and comparative algorithms

#### 2.4.1 PSNR

An adaptive learning mechanism is introduced to dynamically adjust the strategy based on the characteristics of the input image. Both quantitative and qualitative evaluation methods were developed, with the Peak Signal-to-Noise Ratio (PSNR) used to assess the enhancement effect (Wang et al., 2004), as shown in Equation 10. Higher PSNR values indicate lower image distortion, consequently, better quality. Generally, a PSNR greater than 30 dB is considered to signify high reconstruction quality. Equation 9 represents the formula for calculating the Mean Squared Error (MSE) (Jobson et al., 1997).


(9) $$MSE=\frac{1}{mn}\sum_{i=0}^{m-1}\sum_{j=0}^{n-1}{\left(I\left(i,j\right)-K\left(i,j\right)\right)}^{2}$$

Where *MSE* is Mean Square Error; *I (i, j)* and *K (i, j)* are the pixel values of the original and processed image respectively. *m* and *n* are the width and height of the image.


(10) $$\text{PSNR}=10\log_{10}(\frac{MAX^{2}}{MSE})$$

Where *MAX* is the maximum pixel value of the image.

#### 2.4.2 RGB and HLS change laws

In the field of image processing and computer vision, image features are traditionally extracted from the RGB (Red, Green, Blue) channels (Hu et al., 2021; Yan et al., 2022). However, in practical applications particularly in tasks such as object detection, image segmentation, and color correction the HLS (Hue, Lightness, Saturation) color model is often more aligned with human visual perception. This alignment makes color feature extraction more intuitive and effective, especially when interpreting color based scene information. In this study, the RGB and HLS channel profiles were plotted for different soybean rows, arranged from left to right, corresponding to the direction of increasing light intensity across the image. Each channel was statistically analyzed along this orientation to assess how varying illumination conditions affect image representation. The variations in the RGB channels primarily reflect contrast changes in the enhanced images, while changes in the HLS channels more accurately represent perceived color differences from a human visual standpoint. This dual channel analysis facilitates a more comprehensive understanding of how light intensity influences image features, thereby supporting more accurate and robust image detail recognition in subsequent processing stages.

## 3 Result and discussion

### 3.1 Illumination compensation parameterization

In the experiment, light intensity measurements were taken at each growth stage of soybean. Based on variation in light intensity, polynomial regression equations were constructed to systematically study examine the effects of height difference, solar elevation angle, and soybean location on the response variable light intensity, as shown in **Table 1**. Initially, the height difference and solar elevation angle were employed as the primary independent variables, while six distinct soybean positions (30 cm, 60 cm, 90 cm, 120 cm, 150 cm, and 180 cm) were treated as supplementary independent variables, forming a comprehensive set of inputs. Based on this expanded set, linear regression was applied to model the light intensity y at each position, thereby generating corresponding regression equations. These equations, composed of regression coefficients and an intercept term, can be used to predict light intensity at various positions under different combinations of height difference, solar elevation angle, and soybean location, thereby revealing the complex relationships between the independent and response variables.

**Table 1:** Height difference and solar elevation angle of soybean and corn at different fertility periods.

Serial	Test time	Maize growth stage	Soybean growth stage	Altitude difference (cm)	Solar altitude angle
1	6.20	Three-leaf stage	Three-leaf stage	5	33.8°
2	7.15	Seven-leaf stage	flowering period	23	30.4°
3	7.30	heading period	podding stage	113	27.0°
4	8.20	tage of milky ripeness	seed filling period	101	21.4°
5	9.30	maturity	maturity	100	13.9°

**Figure 4:**
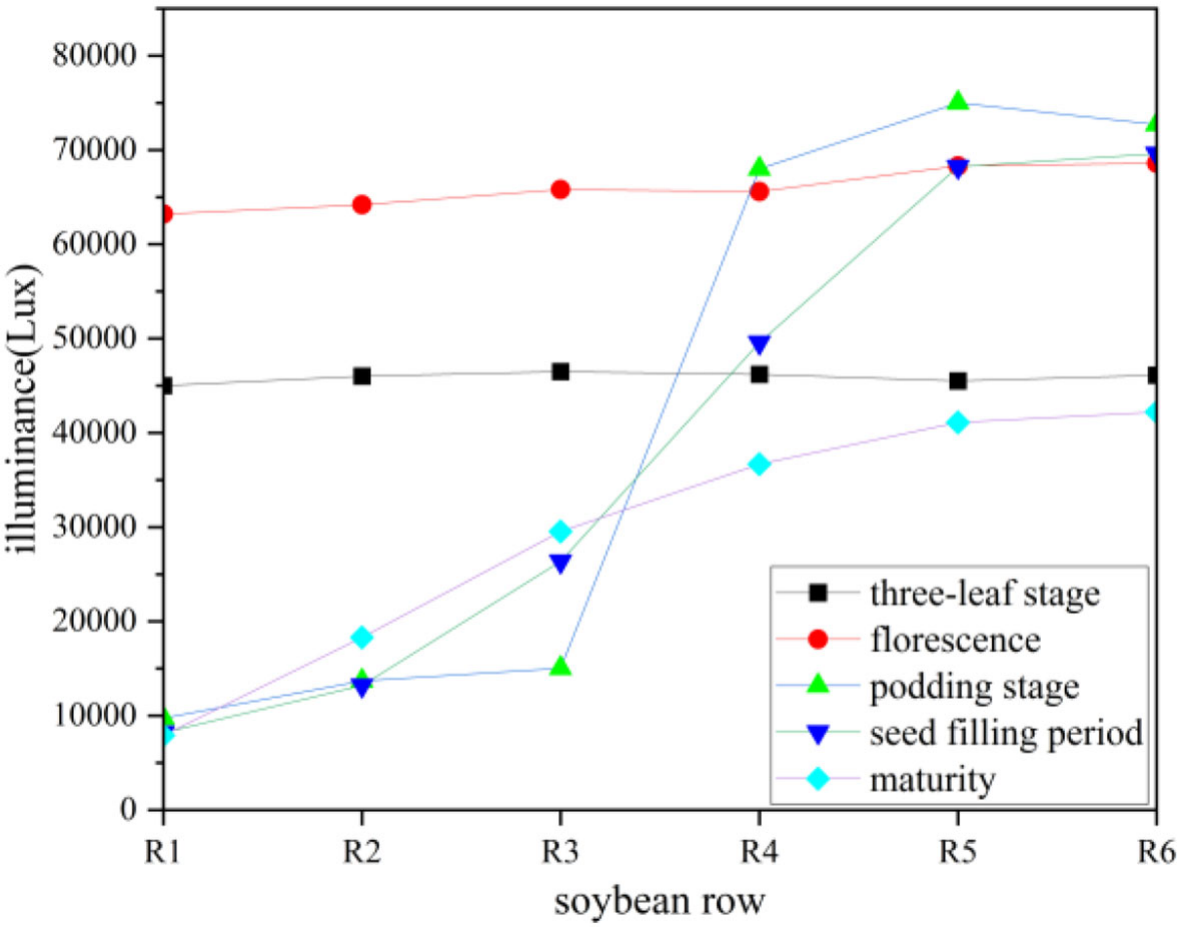
The light intensity variation in the soybean strip at different growth stages.

By applying the regression equation, predicted light intensities for different locations can be calculated, providing a theoretical foundation for experimental design, model validation, and result prediction. This approach offers robust scientific support for further quantifying and understanding the multivariable effects observed in the experiment. As shown in **Figure 4**, the x-axis represents the soybean row numbers, arranged from south to north, while the y-axis denotes light intensity. Light intensity variations for five soybean growth stages the three-leaf stage, flowering stage, podding stage, seed filling period, and maturity stage are plotted. To analyze light intensity variations across these stages, regression analysis was employed to fit the collected data. By selecting an appropriate regression model, the optimal functional form for light intensity changes over time was determined. The least squares method was used to optimize the model parameters, ensuring that the fitted curve accurately represents the light intensity variations along the soybean rows at each growth stage. This fitting result provides insights into the spatial distribution of light intensity, thereby facilitating an analysis of light exposure across the experimental field.

The inputs to the polynomial regression equation are altitude difference, solar altitude angle, and position within the soybean row, denoted by *h*, θ , and l , respectively, and the output is light intensity, denoted by *y*, the R² value of the fitting equation reached 91%. as shown in Equation 11. The Coefficient values for each item are represented by $a_{0}$ - $a_{9}$ , as shown in **Table 2**.

**Table 2:** Coefficient values and definitions used in Equation 11 and Equation 12.

Parameter	Value	Description
$a_{0}$	3102448	Intercept term (constant)
$a_{1}$	-61307	Coefficient for height difference h
$a_{2}$	-8220	Coefficient for solar elevation angle *θ*
$a_{3}$	-515	Coefficient for soybean row position *l*
$a_{4}$	235	Coefficient for $h^{2}$
$a_{5}$	883	Coefficient for ℎ·*θ*
$a_{6}$	16	Coefficient for *θ·l*
$a_{7}$	-2301	Coefficient for *θ* ^2^
$a_{8}$	6	Coefficient for ℎ·*l*
$a_{9}$	-0.334	Coefficient for *l* ^2^


(11) $$\text{y=}a_{0}+a_{1}\text{h + }a_{2}\text{ θ +}a_{3}\;l+a_{4}\;h^{2}\text{ + }a_{5}\;h\text{ θ + }a_{6}\text{ θ }l+a_{7}\;\theta^{2}\text{+ }a_{8}\;h\;l+a_{9}\;l^{2}$$

Combining the statistical equations with the compensation curves and adding the planting direction parameter resulted in the following equations, as shown in Equation 12.

By substituting Equation 11 into Equation 6, and considering that the soybean-maize intercropping in this study is oriented in the north-south direction, a planting direction parameter is introduced into the equation to account for the light intensity variation caused by the planting orientation, resulting in Equation 12.


(12) $$E_{d}=(\begin{array}{c} a_{0}+a_{1}\;h\;+a_{2}\;\theta +a_{3}\;l+a_{4}\;h^{2}+a_{5}\;h\;\theta +\\ \;a_{6}\;\theta l+a_{7}\theta^{2}+a_{8}\;hl+a_{9}\;l^{2}+D_{f}+\text{α} \end{array}){\text{E}}_{0}\text{cos}\left(\delta \right)$$

The image compensation value is denoted as $E_{d}$ , the height difference in the equation is *h*, the solar altitude angle θ , the position l , and δ is the angle between the planting direction and the north-south direction, which can be obtained from the equation for the height difference, the solar altitude angle, the light intensity, and the planting direction angle. The meanings of the letters in the equations are given in **Table 3**.

**Table 3:** Parameter values and definitions in the equation.

Parameter value	Definition
$E_{d}$	The image compensation value
h	Height difference
θ	The solar elevation angle
l	Position within the soybean row
$D_{f}$	The scattered light coefficient
α	Reflectance
${\text{E}}_{0}$	The light intensity of the sun in direct sunlight
δ	The angle between the planting direction and the north-south direction

### 3.2 PSNR indicator results

**Figure 5:**
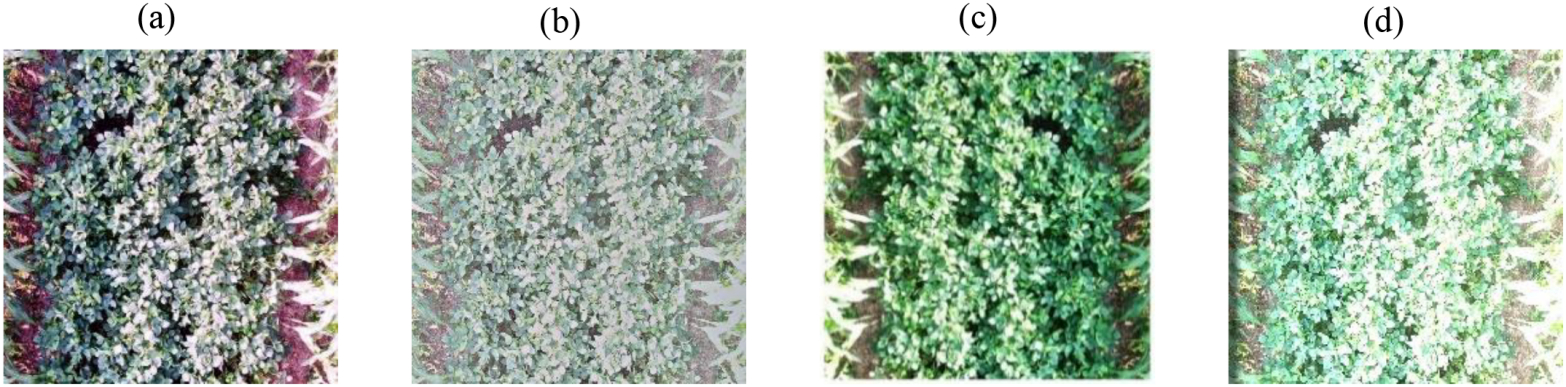
The processing results of the image using different algorithms. **(a)** histogram equalization method, **(b)** Multi-Scale Retinex, **(c)** Gamma function correction, **(d)** ICNet.

In this study, images are processed using the Histogram Equalization method, (MSR), Gamma Correction, and ICNet, Processing of **Figure 1b** using different algorithms. Histogram Equalization redistributes pixel gray levels to achieve a more uniform gray level distribution, as shown in **Figure 5a**. MSR enhances image contrast and detail by estimating and removing the illumination component based on the reflectance-illumination multiplicative model, as shown in **Figure 5b**. Gamma Correction applies a nonlinear transformation to the pixel values to optimize the brightness distribution, as shown in **Figure 5c**. Furthermore, illumination compensation is performed using a light intensity curve variation equation to ensure uniform image brightness, as shown in **Figure 5d**.

In intercropped images, it is assumed that humans extract structural information, leading to the introduction of an alternative complementary framework for quality assessment based on the degradation of such information (Wang et al., 2004). The PSNR was calculated for images processed by several algorithms. A total of 50 images were randomly selected, with 10 images chosen from each growth stage, and processed using various algorithms. Compared to the original image, the PSNR values for the histogram equalization method, Multi-Scale Retinex, and Gamma function correction were all below 30 dB. However, after brightness compensation with ICNet, the PSNR reached 40.79 dB, indicating that the enhanced image quality is excellent, as shown in **Table 4**.

**Table 4:** PSNR values for different algorithms.

Pictures after processing	PSNR(dB)
histogram equalization method	27.76
Multi-Scale Retinex	28.36
Gamma function correction	28.39
ICNet	40.79

### 3.3 RGB variation with different algorithms

**Figure 6:**
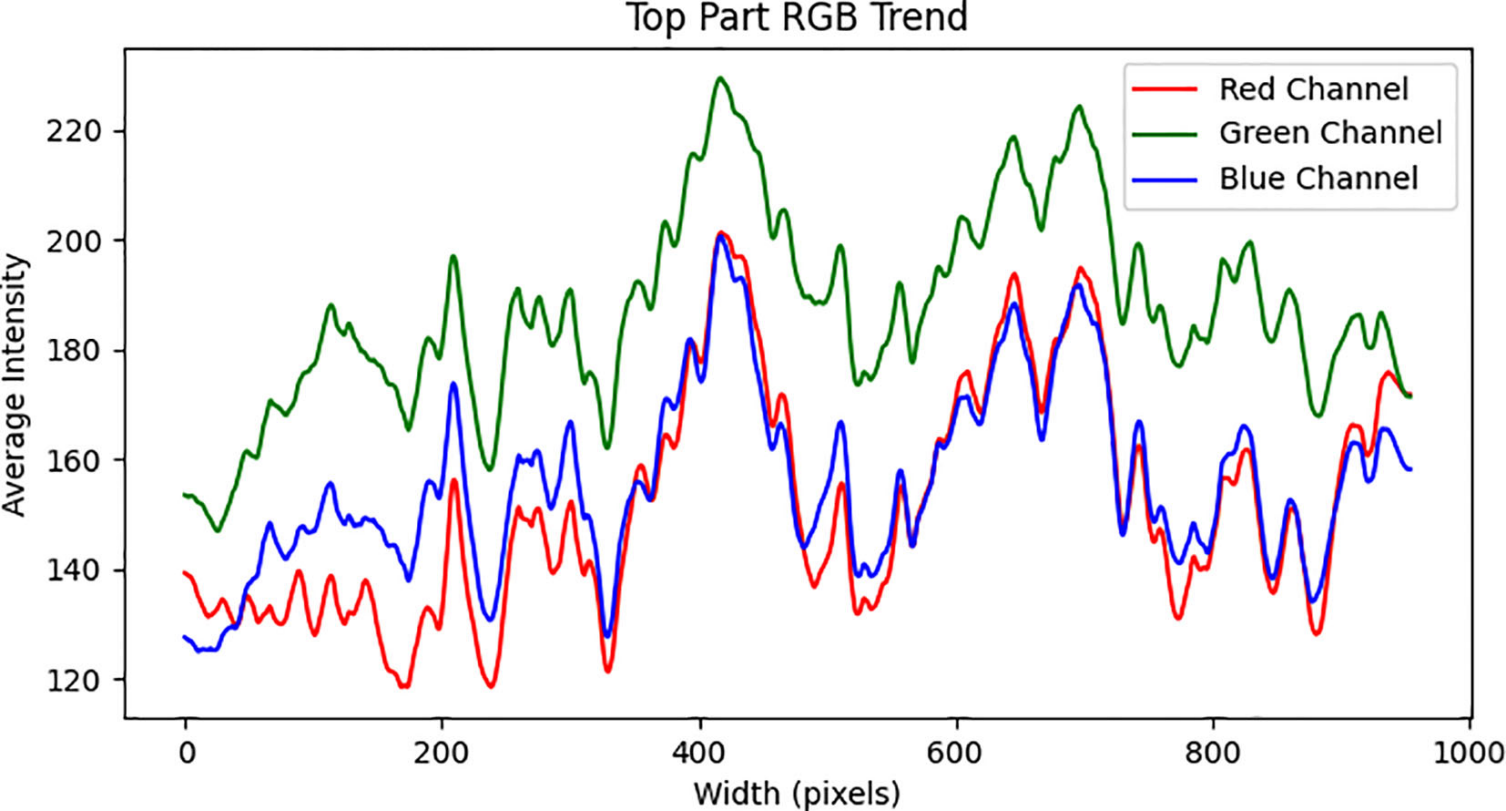
The RGB channel values of the soybean canopy image along the horizontal centerline change from left to right.

Accurate color recognition is critical for image based analysis; however, environmental light variability can significantly compromise its accuracy. Therefore, selecting an appropriate radiometric correction method is essential when operating under varying lighting conditions (Wang et al., 2024). In agricultural settings, changes in ambient illumination can directly affect the detection of vegetation indices, as image-based sensors depend heavily on color channel data to extract meaningful morphological and physiological features (Cardenas-Gallegos et al., 2025). The pod-setting stage of soybean coincides with the period when the height difference between maize and soybean plants reaches its maximum. This stage is also associated with the greatest variation in light intensity over the soybean canopy due to differential shading. In order to assess the performance of various image processing algorithms, three representative images were randomly selected from each phenological stage for comparative analysis. This image was processed using multiple enhancement algorithms, and the RGB and HLS channel responses were examined. The analysis was conducted along the horizontal centerline of the image, with the left side representing the direction closer to the sun (south), and the right side being further away (north). As shown in **Figure 6**, the RGB channel intensities in the soybean strip image demonstrated an increasing trend from left to right (south to north), indicating a gradual rise in overall brightness. Among the three RGB channels, the green channel consistently exhibited higher intensity values compared to the red and blue channels. This dominance of the green component suggests that no significant color shift occurred, and the increase in brightness was relatively uniform across the image. Although the overall trend in all channels was upward, local fluctuations were observed within each channel, reflecting spatial variation in reflectance characteristics across the soybean strip. This implies that intercropping alters the phenotypic appearance of soybeans at different positions due to variable light distribution. On average, red and blue channel intensities ranged from 120 to 200, while the green channel ranged from 150 to 230, further emphasizing the dominant role of green spectral reflectance. The observed left to right gradient in brightness primarily driven by the green channel-demonstrates the importance of addressing directional lighting effects during image analysis in intercropped systems.

**Figure 7:**
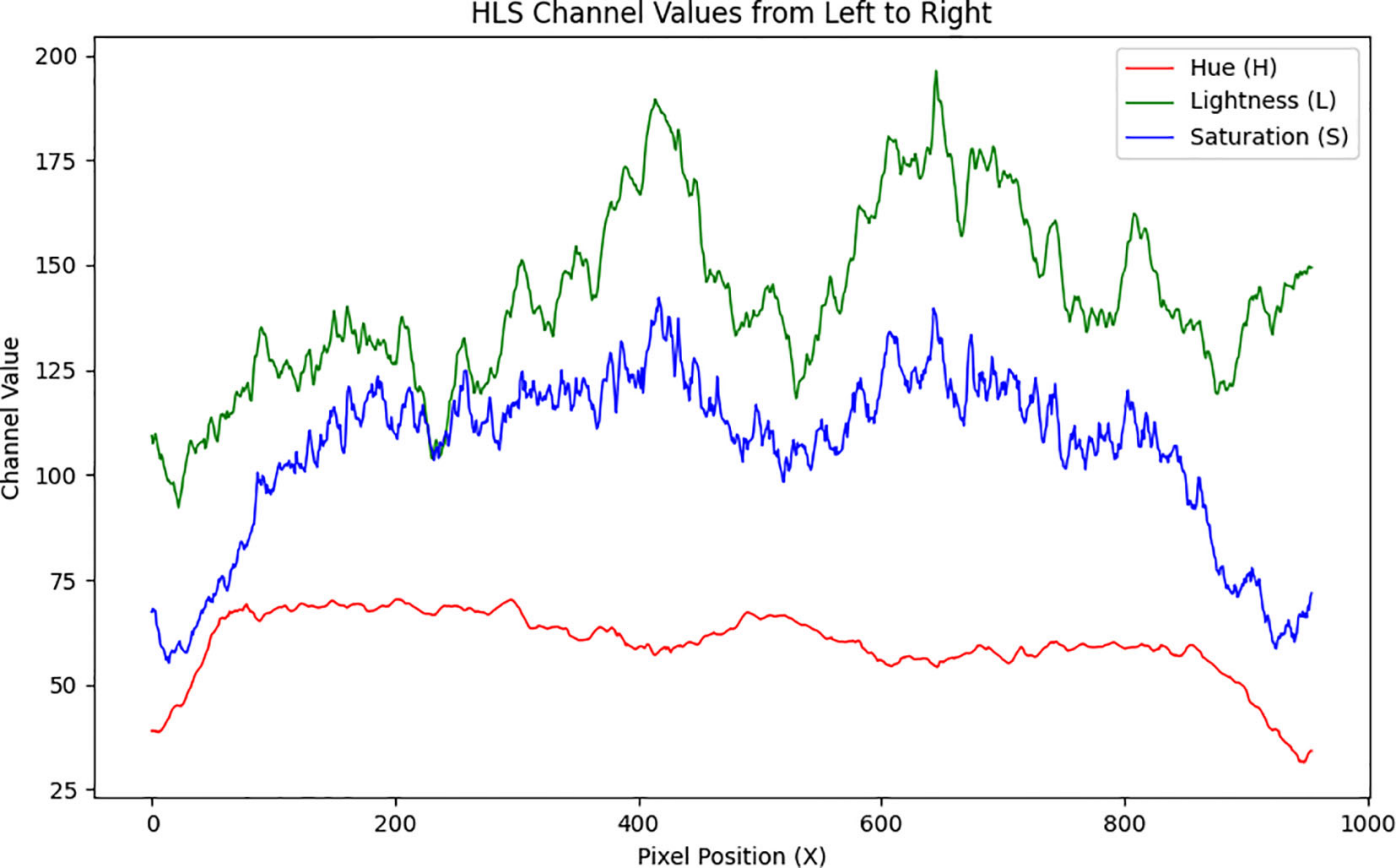
The HLS channel values of the soybean canopy image along the horizontal centerline change from left to right.

As shown in **Figure 7**, the variations in the HLS channels (Hue, Lightness, Saturation) of the soybean strip from south to north reveal that overall brightness exhibits an increasing trend, corresponding to the image transitioning from dark to bright. The hue shows a slight downward trend, corresponding to that the color remains consistent from left to right with no noticeable color shift. This minor change in hue may be attributed to subtle color adjustments in certain regions caused by certain in lighting conditions within the image. The saturation curve displays fluctuations in color purity, exhibiting a slight upward trend overall. This increase in saturation is likely due to the enhanced brightness, which makes the colors appear more saturated and vivid. The observed fluctuations indicate localized variations in color intensity across the soybean strip.

**Figure 8:**
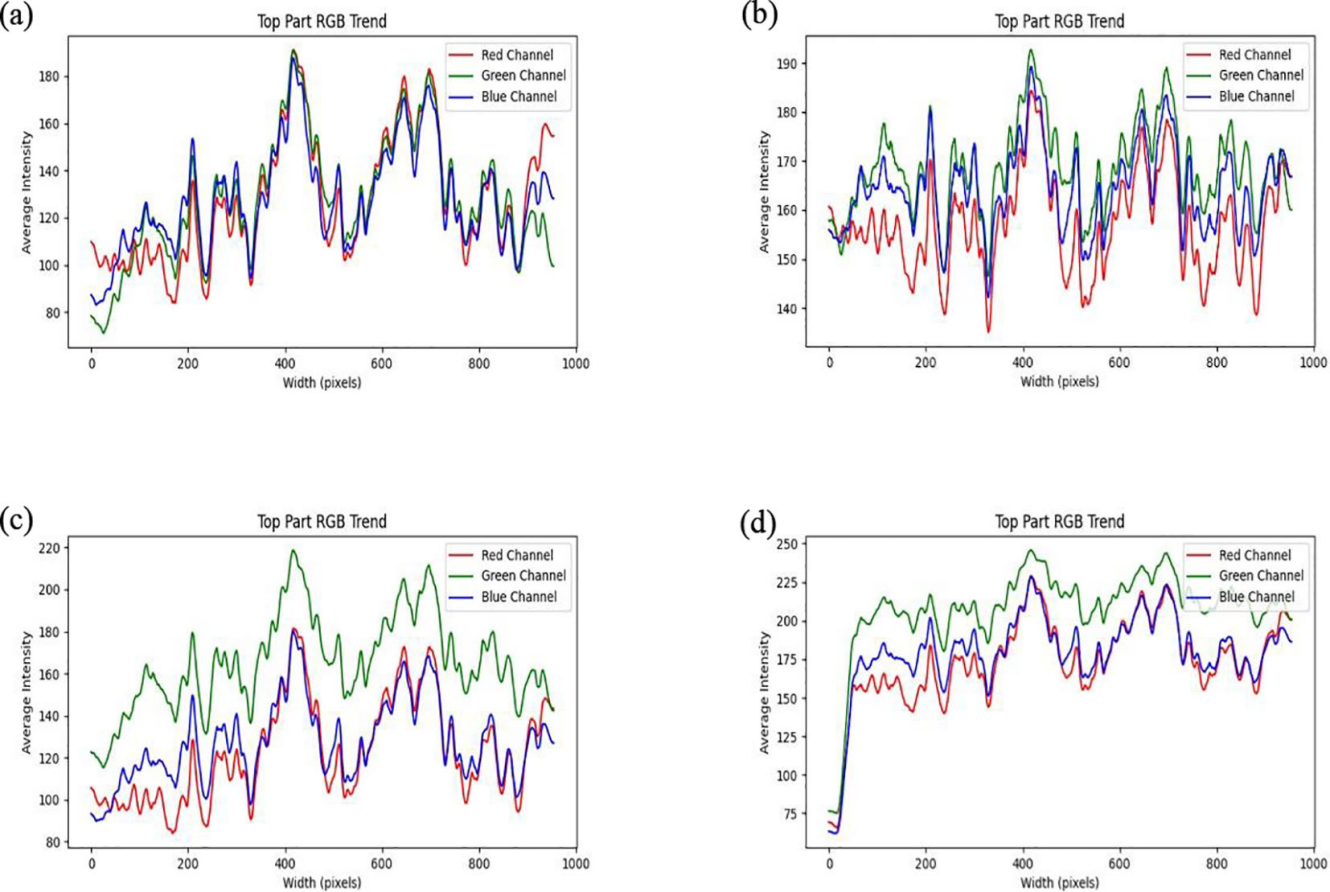
The RGB channel values along the horizontal centerline of the soybean canopy image change from left to right after processing with different algorithms: **(a)** Histogram Equalization Algorithm, **(b)** Multi-Scale Retinex Algorithm, **(c)** Gamma Function Correction Algorithm, **(d)** ICNet Algorithm.

This study compares the trends in RGB and HLS channel variations from left to right in images processed using four different brightness enhancement methods: histogram equalization, MSR, Gamma function correction, and the ICNet algorithm. As shown in **Figure 8a**, the RGB channel values exhibit a consistent decreasing trend, with the overall range reduced to 70-190. Additionally, the fluctuations in values increase, thereby enhancing the image contrast. However, despite the improved contrast, excessive local contrast in certain areas results in detail blurring or loss. Moreover, pronounced color shifts were observed, characterized by an overall purple hue that led to localized saturation and subsequent color distortion. As shown in **Figure 8b**, the fluctuations in RGB channel values significantly decrease, with the variation range reducing from 80 to 50, and all maximum values remaining below 200. This indicates a notable reduction in image brightness, leading to color homogenization across the image. As illustrated in **Figure 8c**, gamma correction effectively maintains overall image brightness. The RGB channel values exhibit a general decline, with a maximum G-channel value of 220 and maximum R and B-channel values of 180. However, the variation trends remain consistent with those of the original image, suggesting that the overall brightness decreases without altering the color relationships or contrast dynamics. A limitation of gamma correction is its linear brightness adjustment, which may be ineffective in enhancing details in darker regions under complex lighting conditions. In contrast, **Figure 8d** demonstrates that the ICNet algorithm leads to a substantial increase in RGB channel values, with all values exceeding 150, indicating a significant overall brightness enhancement. Additionally, the reduced RGB value fluctuations result in more uniform color and brightness distribution, leading to smoother color transitions and reduced differences between bright and dark areas. Compared with other image enhancement methods, the ICNet algorithm effectively increases the overall brightness while simultaneously reducing RGB fluctuations, thereby producing smoother color transitions and more visually balanced images.

**Figure 9:**
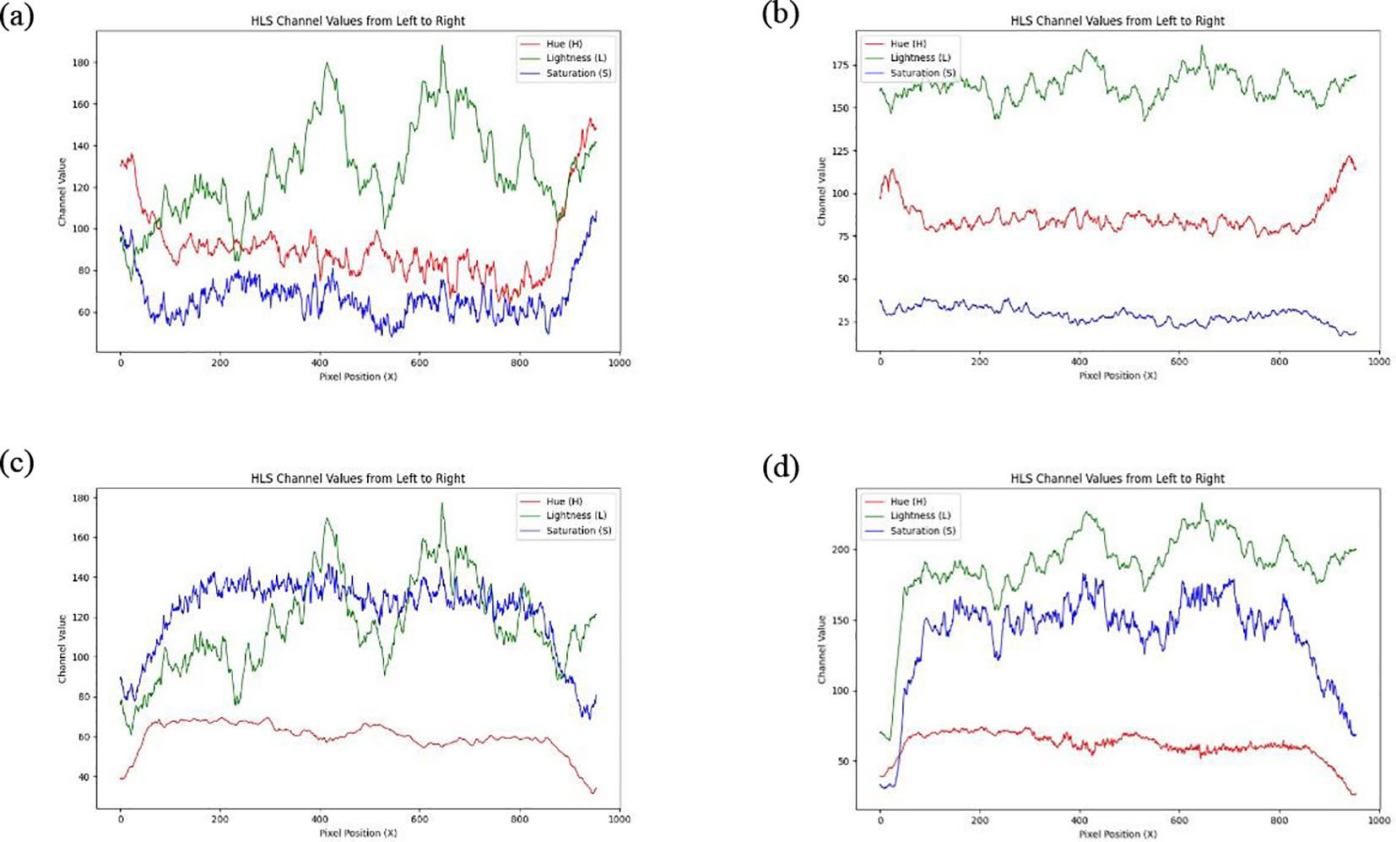
The HLS channel values along the horizontal centerline of the soybean canopy image change from left to right after processing with different algorithms: **(a)** Histogram Equalization Algorithm, **(b)** Multi-Scale Retinex Algorithm, **(c)** Gamma Function Correction Algorithm, **(d)** ICNet Algorithm.

This study evaluates the performance of four image brightness enhancement techniques Histogram Equalization, Multi-Scale Retinex (MSR), Gamma Correction, and the ICNet algorithm within the HLS color space. As shown in **Figure 9a**, histogram equalization results in significant lightness fluctuations, with a maximum value reaching 190, indicating considerable variations in brightness across different regions of the image. While the overall lightness trend shows a slight increase, excessive contrast enhancement in some areas leads to an uneven brightness distribution. The mean saturation remains relatively stable, but the noticeable oscillations imply poor color stability, negatively affecting overall color balance. Additionally, hue values fluctuate between 70 and 140, suggesting that histogram equalization may introduce color distortions, especially during high contrast enhancement. As illustrated in **Figure 9b**, the MSR method provides effective brightness enhancement, with lightness values exceeding 150 and displaying a relatively stable upward trend, improving detail visibility in darker regions. However, saturation drops markedly compared to the original image, with values remaining below 50, indicating a transition toward grayscale that may reduce image vibrancy and contrast between bright and dark regions. Furthermore, the overall increase in hue values (all above 75) reflects a degree of color shift caused by hue modification. In **Figure 9c**, gamma correction demonstrates relatively stable brightness enhancement, although the maximum lightness value decreases from 200 to 180, indicating an overall reduction in image brightness. Brightness fluctuations, however, increase from 100 to 120, suggesting a greater contrast between bright and dark areas, which may result in detail loss in some regions. Meanwhile, saturation increases from 130 to 140, and its fluctuation narrows from 80 to 60, implying that the image colors become more vivid, though with reduced tonal diversity, potentially affecting perceived contrast. The hue values and their fluctuations remain largely consistent with the original image, indicating no significant alteration in overall color composition. As depicted in **Figure 9d**, the ICNet algorithm exhibits the smallest brightness fluctuations among the four methods, demonstrating superior stability under complex lighting conditions while preserving image details. Notably, the brightness in the left portion of the image increases from 125 to 175, signifying a substantial enhancement. Saturation surpasses 150, reflecting improved color intensity without sacrificing contrast perception. Additionally, hue values and fluctuations remain unchanged compared to the original image, confirming the preservation of the original color composition. The uniform brightness distribution and enhanced color vividness, particularly in shadowed regions, highlight the ICNet algorithm’s superior performance in maintaining image quality and visual consistency.

### 3.4 Standard deviation variation

Standard deviation is a commonly used statistic to describe the distribution characteristics of data. In image processing and analysis, it reflects the image’s brightness, color variations, and noise characteristics. A larger standard deviation indicates stronger texture, edges, or noise. Ten images were randomly selected from each growth stage, for a total of 50 images, and processed using various algorithms. Based on the previous findings in the RGB and HLS channels, it was observed that the leftmost 1/10 and the rightmost 1/10 of the image correspond to the dividing line between soybean and corn, where significant changes in the RGB and HLS channels occur. In subsequent calculations of standard deviation and peak-to-peak values, these two sections are excluded from the analysis.

**Figure 10:**
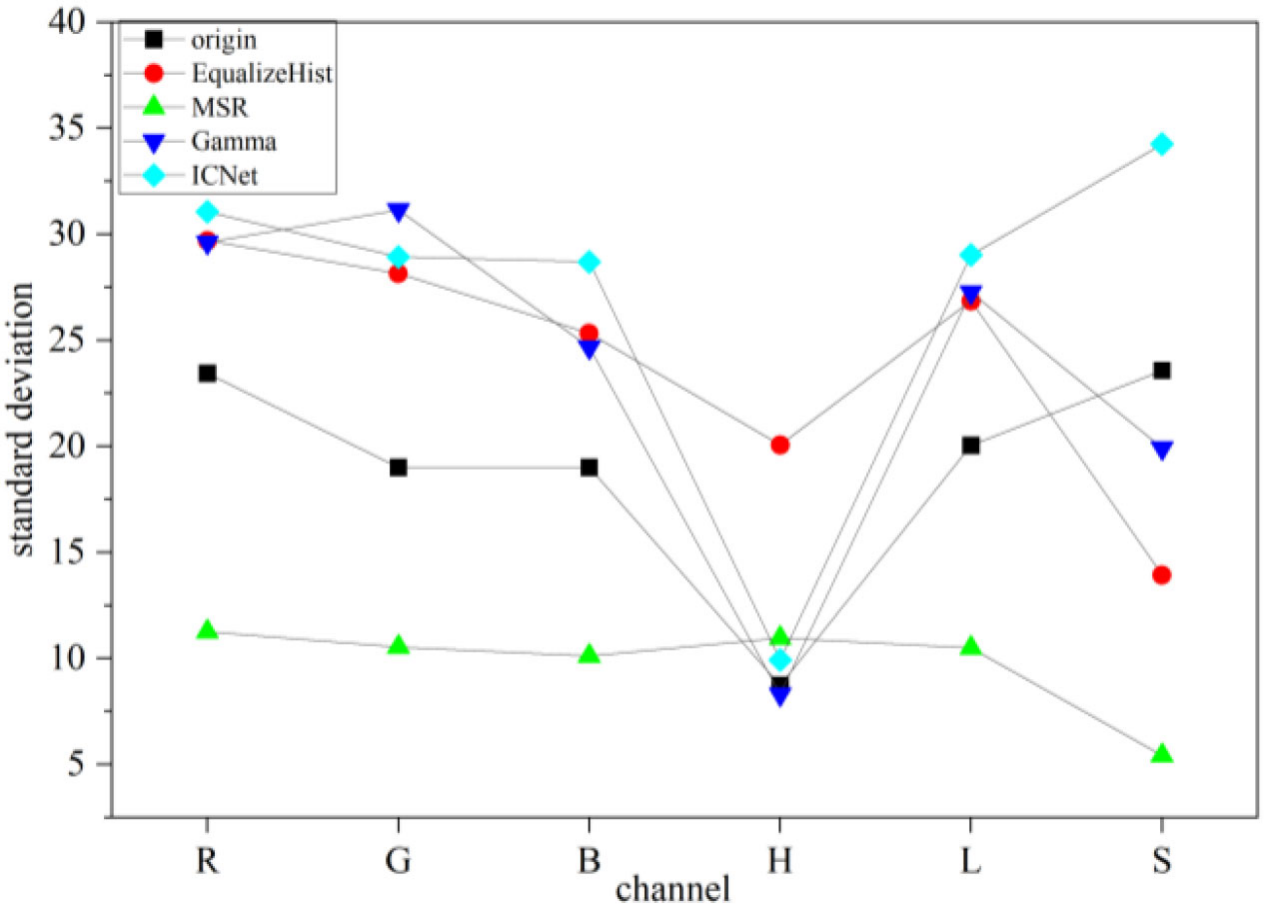
Variation in the standard deviation of the RGB and HLS channel values of the soybean canopy image after processing with different algorithms.

As shown in **Figure 10**, the ICNet algorithm demonstrates superior performance compared to traditional methods. While histogram equalization introduces a slight decrease in the H channel, ICNet effectively enhancing image details by increasing the standard deviation without significantly amplifying noise or artifacts. During detail enhancement, ICNet achieves a smooth increase in standard deviation, mitigating the risk of unnatural sharpening effects. Compared to multi-scale processing, ICNet exhibits a smaller deviation in the H channel, striking a better balance between local detail enhancement and overall image consistency. Additionally, ICNet reduces the common noise amplification issues seen in MSR processing, ensuring more stable standard deviation performance. Furthermore, in contrast to gamma correction, ICNet significantly improves the S channel’s stability while maintaining consistent values in other channels. This refined brightness optimization enhances contrast and preserves image details more effectively, avoiding the excessive darkening or lightening that often occurs with gamma correction. Compared to traditional methods, the ICNet algorithm demonstrated lower standard deviation values across key channels. Specifically, in the green (G) channel, the standard deviation decreased from 18.5 (MSR) to 12.1 with ICNet, representing a 34.6% reduction. Similarly, the hue (H) channel’s variation decreased by 27.3%, indicating improved chromatic consistency. These quantitative results, illustrated in **Figure 10**, substantiate the enhanced brightness uniformity and reduced noise fluctuations achieved by the proposed method, supporting its effectiveness under uneven illumination conditions.

### 3.5 Comparative analysis and method evaluation

To comprehensively evaluate the performance of the proposed ICNet algorithm, a comparative analysis was conducted against three conventional image enhancement methods: histogram equalization, multi-scale Retinex (MSR), and gamma correction. The comparison was based on multiple metrics, including PSNR, RGB and HLS channel profiles, and standard deviation distribution. Among the four methods, ICNet achieved the highest PSNR value (40.79 dB), significantly outperforming the other three methods, which remained below 30 dB. As shown in **Figures 8** and **9**, ICNet enhanced image brightness uniformly across the soybean canopy, with RGB channel values consistently above 150 and reduced channel fluctuations, indicating smoother transitions and improved visual consistency. In contrast, histogram equalization exhibited strong contrast enhancement but resulted in local overexposure and color distortion. MSR effectively enhanced shadow regions but introduced visible hue shifts and reduced color saturation. Gamma correction preserved natural color relationships but showed limited enhancement in underexposed areas and was less effective in handling brightness imbalance. The primary advantage of ICNet lies in its ability to perform spatially adaptive brightness correction by leveraging crop height difference, solar elevation angle, and planting orientation. This physics informed compensation strategy enables uniform lighting enhancement, particularly in images affected by crop shading. Additionally, ICNet maintains low standard deviation in green (G) and hue (H) channels, which reflects better image stability and noise control. However, the proposed method also has limitations. ICNet is based on polynomial regression models fitted from field-acquired data. This dependence on manually measured canopy parameters and solar angles limits its scalability and adaptability to other cropping systems or image acquisition conditions. Furthermore, as a rule based method, ICNet lacks the capacity to autonomously learn from new data, making it less flexible than deep learning based approaches in highly dynamic or heterogeneous environments. Future improvements may focus on integrating ICNet with data driven models, such as convolutional neural networks (CNNs), or replacing static regression equations with adaptive learning modules, to enhance generalizability and robustness in broader agricultural applications. Although manual measurements were used in this study, future implementations can leverage UAV photogrammetry or LiDAR to automate plant height and shading parameter acquisition. Preliminary deployment tests showed that ICNet inference time was under 0.15 s per image on a standard GPU, indicating its feasibility for real time field applications.

## 4 Conclusion

This study developed an illumination compensation algorithm (ICNet) tailored for image enhancement in maize–soybean intercropping systems. By modeling the relationship between crop height difference, solar elevation angle, and canopy light intensity, a polynomial regression-based compensation framework was established and applied to correct uneven lighting across soybean canopy images. Experimental results demonstrated that ICNet achieved a PSNR of 40.79 dB, significantly outperforming traditional methods such as histogram equalization (27.76 dB), multi-scale Retinex (28.36 dB), and gamma correction (28.39 dB). Additionally, RGB and HLS channel analysis showed that ICNet effectively increased brightness uniformity while reducing channel fluctuations green channel values exceeded 150, and brightness in shadowed regions improved from 125 to 175. Standard deviation analysis confirmed enhanced image consistency, with up to 35% reduction in variation across key color channels. Compared to conventional techniques, ICNet not only improves visual clarity but also preserves color fidelity and suppresses noise, providing a more robust foundation for accurate image based crop trait extraction in precision agriculture. These findings validate the method’s practical value for improving the reliability of visual analysis under complex intercropping light conditions. Future work will focus on enhancing the generalizability of the model through adaptive or learning based modules to accommodate diverse crop architectures and lighting environments. The proposed ICNet algorithm outperformed traditional methods in PSNR and HLS stability, but its performance under extreme overcast or strong shadow conditions requires further validation.

## Data Availability

The original contributions presented in the study are included in the article/supplementary material. Further inquiries can be directed to the corresponding author.
